# Genome-wide analysis of carotid plaque burden suggests a role of IL5 in men

**DOI:** 10.1371/journal.pone.0233728

**Published:** 2020-05-29

**Authors:** Janne Pott, Frank Beutner, Katrin Horn, Holger Kirsten, Kay Olischer, Kerstin Wirkner, Markus Loeffler, Markus Scholz

**Affiliations:** 1 Institute for Medical Informatics, Statistics and Epidemiology, University of Leipzig, Leipzig, Germany; 2 LIFE Research Center for Civilization Diseases, University of Leipzig, Leipzig, Germany; 3 Leipzig University Medical Center, IFB Adiposity Diseases, University of Leipzig, Leipzig, Germany; 4 Heart Center Leipzig, Leipzig, Germany; Brigham and Women's Hospital and Harvard Medical School, UNITED STATES

## Abstract

**Background:**

Carotid artery plaque is an established marker of subclinical atherosclerosis with pronounced sex-dimorphism. Here, we aimed to identify genetic variants associated with carotid plaque burden (CPB) and to examine potential sex-specific genetic effects on plaque sizes.

**Methods and results:**

We defined six operationalizations of CPB considering plaques in common carotid arteries, carotid bulb, and internal carotid arteries. We performed sex-specific genome-wide association analyses for all traits in the LIFE-Adult cohort (n = 727 men and n = 550 women) and tested significantly associated loci for sex-specific effects. In order to identify causal genes, we analyzed candidate gene expression data for correlation with CPB traits and corresponding sex-specific effects. Further, we tested if previously reported SNP associations with CAD and plaque prevalence are also associated with CBP. We found seven loci with suggestive significance for CPB (p<3.33x10^-7^), explaining together between 6 and 13% of the CPB variance. Sex-specific analysis showed a genome-wide significant hit for men at 5q31.1 (rs201629990, β = -0.401, *p* = 5.22x10^-9^), which was not associated in women (β = -0.127, *p* = 0.093) with a significant difference in effect size (*p* = 0.008). Analyses of gene expression data suggested *IL5* as the most plausible candidate, as it reflected the same sex-specific association with CPBs (p = 0.037). Known plaque prevalence or CAD loci showed no enrichment in the association with CPB.

**Conclusions:**

We showed that CPB is a complementary trait in analyzing genetics of subclinical atherosclerosis. We detected a novel locus for plaque size in men only suggesting a role of IL5. Several estrogen response elements in this locus point towards a functional explanation of the observed sex-specific effect.

## Introduction

Atherosclerosis is a chronic disease characterized by arterial lumen reduction due to arterial wall thickening and development of plaques. Major risk factors are high cholesterol, age, sex, smoking and obesity [1]. As atherosclerosis has a long pre-clinical phase, non-invasive methods can be used for early detection. Those methods can be used to identify people who are at high risk of a cardiovascular event [2], and thus offers a means of prevention [3]. B-mode ultrasound measurement of the carotid arteries is one of these non-invasive methods with which the carotid intima media thickness (cIMT) and carotid plaques sizes can be measured [4].

Genetic predisposition also contributes to atherosclerosis risk: The latest meta-analyses with 34,541 cases of coronary artery disease (CAD) and 261,984 controls reported 169 genetic risk loci with genome-wide significance (p<5x10^-8^) [5]. For carotid plaques, 14 loci were described with at least suggestive significance (p<1x10^-6^) [6]. Both CAD and carotid plaques share genetic risk loci, such as the *9p21* locus [5–7], and are genetically correlated [6].

Male sex is an independent risk factor of several traits and diseases including atherosclerosis, although the underlying mechanisms are only partly understood [8,9]. Recent genome-wide analyses detected sex-specific effects across biomarkers [10,11], especially for steroid hormones [11]. It is yet unclear what causes the difference of effects, e.g. whether it is related to the role of the underlying enzyme in steroid metabolism, or due to hormone response elements within the genome controlling gene expression. Mendelian Randomization has shown male-specific causal effects of cortisol and androstenedione, and female-specific causal effects of dehydroepiandrosterone sulfate (DHEA-S) on CAD [11]. For cIMT, Dong et al. [12] reported SNP-sex interaction for two genetic loci.

Genetics of atherosclerosis onset and progression / severity could be different. Zeller et al. [13] only detected one hit for Gensini score [14] as a marker of cardiovascular disease severity. A recent GWAS on myocardial infarction risk among patients with stable CAD revealed a limited number of loci and detected pronounced differences of effects on CAD onset and MI (personal communication).

To the best of our knowledge there is only one published study on genetics of carotid plaque sizes. Della-Morte et al. [15] analyzed the interaction of SNPs and smoking on carotid plaque burden (CPB) in 665 people of Caribbean Hispanic ancestry, followed by replication analyses in 264 people of the same ancestry. While they detected two significant SNP-smoking interactions, they did not describe any genome-wide significant loci. However, this study was far less powered than studies of CAD or plaque prevalence making further efforts to understand genetics of atherosclerosis progression a worthwhile endeavor.

Here, we conducted a genome-wide association study (GWAS) of six CPB traits in 1277 Europeans, considering both the absolute sizes of and the relative lumen reductions by the plaques. We aimed to discover genetic markers for CPB and estimate the heritability of carotid plaque sizes. Since sex is one of the strongest risk factors, we attempted to find SNP-sex interactions at candidate loci both on genome and transcriptome level. We also analyzed known genetic CAD and carotid plaque loci for association with our CPB traits to check for a genetic overlap with these traits.

## Material and methods

### Cohort description

The LIFE-Adult cohort is a population-based study with a focus on civilization diseases such as type 2 diabetes and subclinical forms of cardiovascular diseases. 10,000 individuals were recruited in the city of Leipzig in an age- and sex-stratified manner. The baseline examination was conducted from August 2011 to November 2014. Details can be found elsewhere [16]. LIFE-Adult meets the ethical standards of the Declaration of Helsinki and has been approved by the Ethics Committee of the Medical Faculty of the University of Leipzig (Registration Number 263-2009-14122009). Written informed consent including agreement with genetic analyses was obtained from all participants.

### Carotid ultrasound and plaque assessment

A standardized carotid ultrasound was applied to all participants of LIFE-Adult, except those with cervical spine disorder or wounds at the scanning area. High-resolution B-mode ultrasound images of carotid vessels were acquired using the GE Vivid ultrasound platform with a 12.0-MHz linear-array transducer (GE-Healthcare). During the assessments, subjects were in the supine position while trained study nurses scanned four anatomical regions for carotid plaques: *common carotid artery* (CCA), *carotid bulb* (Bulb) and proximal parts of the *internal carotid artery* (ICA) and the *external carotid artery* (ECA) on both sides of the neck. Details can be found elsewhere [7].

Carotid artery plaque was defined according to the American Society of Echocardiography Intima-Media Thickness Task Force [4]. In detail, a lesion was counted as plaque if one of the following three criteria was met:

1. echogenic thickening of intimal reflection that extends into the arterial lumen at least 0.5 mm;
2. swell of more than 50% of the surrounding intima-media thickness;
3. thickness of intima and media >1.5 mm.

Plaque presence was documented as ‘present’ or ‘absent’ or ‘missing’ if the quality of the image was insufficient.

While recruitment was still in process, 1300 samples were selected for a detailed assessment of the sizes of plaques in CCA, Bulb and ICA (ECA was neglected due to lower image qualities). The selection criterion was at least one plaque present in any region and genetic data available (first round of genotyping), with preference for samples with two or more plaques (n = 1804 with plaque present and genetic data available). Using cross-sectional images, total plaque area and total lumen area were determined. We defined and analyzed six traits of carotid plaque burden (CPB):

- CPA_max: area of the largest plaque detected at any of the six regions
- CPA_sum: sum of plaque areas at all six regions
- CPA_mean: mean area of detected plaques
- CPS_max: maximal degree of stenosis (plaque area / lumen area) at any of the six regions
- CPS_sum: sum of stenosis at all six regions
- CPS_mean: mean degree of stenosis

Due to the complex and time-consuming measuring in six regions, the sample selection for the subsequent rounds of genotyping was not updated.

### Genotyping

We genotyped 7,838 participants of LIFE-Adult using the genome-wide SNP array Affymetrix Axiom CEU1 and the software Affymetrix Power Tools (version 1.20.6). Quality control (QC) of the genotype calling was performed following Affymetrix’s best practice steps [17].

Sample QC included dish-QC (<0.82), sample call rate (<97%), sex-mismatch, problematic relatedness (e.g. sample mix-up) and irregularities of XY intensity plots (e.g. XXY samples filtered for gonosomal analyses). We assessed genetic heterogeneity with principal component analyses and filtered outliers (>6 SD in any of the first 10 principal components) for further analysis.

SNP QC comprised SNP call rate, parameters of cluster plot irregularities according to Affymetrix’s recommendations, violation of Hardy-Weinberg equilibrium (p<10^−6^ in an exact test for autosomes, p<10^−4^ for chromosome X with women only [18]) and plate association (p<10^−7^).

After sample and SNP QC, 7669 samples and 532,676 SNPs were imputed on the reference 1000 Genomes Phase 3 [19], software SHAPEIT [20] v2r900 for prephasing and IMPUTE2 [21] v2.3.2 for genotype estimation. For chromosome X, the same settings were used.

### Statistical analysis

All six CPB traits were log transformed to approximate normal distributions (see S1 Fig). If not stated otherwise, statistical analyses were performed with the software R [22]. We tested the correlation between CBPs and cIMT using spearman’s rank correlation. Univariate and multivariate associations of known risk factors of atherosclerosis (sex, age, BMI, hypertension (defined by positive anamnestic information, specific treatment or systolic blood pressure>140mmHg), diabetes type 2 (defined by positive anamnestic information, specific medication or HbA1c≥6.5%), smoking status (never vs. former vs. active smokers), and statin treatment) on CPBs were calculated using linear regression. We also analyzed the correlation of CPBs with total cholesterol, high and low density cholesterol (HDLC and LDLC, respectively), adjusting for sex and statin treatment.

#### Heritability

To estimate the heritability of carotid plaque burden we used the tool GCTA (version 1.92.0beta3) [23]. We calculated a genetic relationship matrix for each autosomal chromosome and estimated the variance explained by the SNPs of each chromosome by separate mixed model analyses. Finally, we combined the results assuming the autosomal chromosomes as statistically independent:

$$h^{2}=\sum_{i=1}^{22}h_{i}^{2};\;SE(h^{2})=\sqrt{\sum_{i=1}^{22}{SE(h_{i}^{2})}^{2}}$$

For each phenotype, we estimated the heritability in three models: unadjusted; adjusted for sex and age only; and adjusted for cardiovascular risk factors (sex, age, BMI, smoking status, statin treatment, type 2 diabetes, and hypertension).

#### Genome-wide association analyses

For each CPB trait, separate genome-wide analyses were performed for all samples (adjustment for sex and age) and for the subgroups of sexes (adjustment for age). All analyses were executed with PLINK 2.0 [24,25] using the additive frequentist model and expected genotype counts. X-chromosomal SNPs were analyzed assuming total X inactivation. We checked for associations with the genetic principal components, but detected none (see S1 Table), therefore, we refrained from adjusting for PCs in our GWAS analyses. We filtered SNPs with MAF<1% or imputation info-score<0.8. Adjusting for the three GWAS, we set the genome-wide and suggestive significance level to *α_gw_* = 1.67×10^−8^ and *α_sug_* = 3.33×10^−7^, respectively. Associations reaching at least suggestive significance in one of the GWAS were considered as “associated” in the following. Associated SNPs were ordered by significance and summarized to loci by building the set of associated SNPs in linkage disequilibrium (LD; r^2^>0.1) or in physical proximity (+/- 500 kb) to the respective lead SNP until all SNPs are assigned. For each locus, a regional association plot was generated. After manual inspection of these plots, we removed one locus showing no support of the lead SNP, as no other variant but the lead SNP was associated at this locus. LD was calculated using LIFE-Adult data for all samples with CPB traits, providing matching LD parameter for association statistics.

We merged all GWAS results and annotated them with the following bioinformatics resources: nearby Ensembl genes (+/-250 kb) [26], comparison with variants reported in the GWAS Catalog (LD r^2^>0.3) [27], expression quantitative trait loci data (LD r^2^>0.3; filtering for eQTLs with *α_cis_* = 0.05 and *α_trans_* = 1×10^−7^ for cis- and trans-eQTLs, respectively) [28–32], and pathways from KEGG, GO, DOSE and Reactome [33,34]. LD to SNPs from GWAS Catalog or eQTL variants were calculated using data from 1000 Genomes Phase 3, Version 5 (2015) for European samples [19].

#### Subsequent analyzes of lead SNPs

We performed difference test [35] for all lead SNPs:

$$Z_{diff}=\left({\hat{\beta }}_{men}-{\hat{\beta }}_{women}\right)/\sqrt{{se}_{men}^{2}+{se}_{women}^{2}},$$

using a Bonferroni-corrected significance level *α_Diff_* = 0.0071, adjusting for 7 lead SNPs.

Furthermore, we analyzed robustness and sex-specificity of our detected loci by comparing the following models: 1) we additionally adjusted for the first ten genetic principal components to test for robustness. 2) We adjusted for cardiovascular risk factors (BMI, smoking status, statin treatment, type 2 diabetes, and hypertension) to analyze possible confounding effects. 3) We used all 16 SNPs in a multivariate model to detect combined effects. Models were analyzed for all individuals and for men and women separately.

For the top locus 5q31.1 and top phenotype CPA_mean, we performed credible set analyses, using 2,245 SNPs within +/-500 kb of the lead SNP rs201629990 [36,37], for all samples and the subset with men and women only, respectively. We used the R-package “gtx” to derive Approximate Bayes Factors (ABF) from the effect estimates and standard errors. Standard deviation priors were chosen depending on effect size ranges (95% range of SNP effects by (2*1.96)): 0.058 for all samples, 0.085 in men, and 0.055 in women. The ABFs were then used to calculate the posterior probability for the variant driving the association signal (e.g. causal variant). We ordered variants by their posterior probability and the calculated for each SNP the cumulative posterior, that is the sum of posterior probabilities of SNP with less than or equal order. The 99% credible sets contain with a 99% cumulative posterior probability the causal variant. To validate our results, we considered the association of SNPs at 5q31.1 with the carotid plaque score (PS), which we analyzed in a previous work [7]. In short, PS is the sum of carotid plaques at CCA and Bulb on both sides of the neck, taking values from 0 to 4. For PS we used a prior of 0.052.

#### Gene expression look-up

To functionally classify the observed SNP-sex interaction at 5q31.1, we analyzed blood gene expression profiles of candidate genes for association with CPBs. For 3,527 LIFE-Adult participants, whole blood was collected in Tempus Blood RNA Tubes (Life Technologies) and relocated to -80°C before further processing. Isolated RNA was processed and hybridized to Illumina HT-12 v4 Expression BeadChips (Illumina, San Diego, CA, USA) and measured on the Illumina HiScan. Raw data of 48,107 gene-expression probes were extracted by Illumina GenomeStudio without additional background correction. Data were further processed within R/Bioconductor. Expression values were log2-transformed and quantile-normalized. Batch effects of expression BeadChips were corrected using an empirical Bayes method [38]. Restricting on subjects with CPBs, a sample size of 935 (537 men, 398 women) was available. Of the 40 candidate genes obtained from eQTL data and nearby gene annotation of the lead SNP, 32 were available with high quality probes in our data. All gene-expressions were adjusted for age, lymphocytes and monocytes.

#### Analysis of known atherosclerosis SNPs

We analyzed SNPs known to be associated with CAD (n = 169 SNPs in van der Harst et al. [5]), carotid plaques or carotid intima media thickness (GWAS catalog search; n = 133 SNPs) [6,7,12,15,39–44]) in more detail to verify possible associations with plaque burden. For CAD, 159 SNPs were available in our data and for one we used a proxy in high LD (r^2^ = 0.993). For the carotid SNPs, 105 were available in our data, and for one we used a proxy (LD r^2^ = 0.732). SNPs were defined as verified for association with plaque burden if they reached at least p<0.05/6 (Bonferroni correction for six phenotypes of plaque burden) and had concordant direction of the effect. To test for enrichment of associated SNPs, we performed a binomial test.

In addition, we tested a polygenetic score (PGS) of CAD for association with CPB traits in men and women separately, and both. Effect weights were obtained from PGS Catalog [45] for 1,7 million variants using data from Inouye et al. [9] (PGS ID: PGS000018). We filtered variants with MAF<1%, imputation info score<0.8 and mismatching alleles in our data, and calculated the PGS as sum of effect weight times genedoses over 1,378,307 SNPs.

## Results

### Impact of non-genetic parameters on plaque burden

Basic characteristics of study subjects are summarized in Table 1. All participants had at least one carotid plaque, with 96% occurring in the carotid bulb. Irrespective of the considered CPB parameter, men had significantly higher plaque burden than women. On average, women had fewer plaques at ACC, bulb and ACI (2.2 plaques in women; 2.9 plaques in men). While the age distribution was the same for men and women (p = 0.127), all other known risk factors of atherosclerosis differed significantly. All CPB traits were highly correlated with each other (spearman ρ between 0.70 and 0.96, p<2x10^-16^, see S2 Fig).

**Table 1:** Characteristics of study subjects.

Parameter		Men	Women	p
Sex		727 (57.0%)	550 (43.0%)	---
Age (years)		64.9 (9.7)	64.2 (9.6)	0.127
BMI (kg/m^2^)		28.1 (4.0)	27.9 (5.0)	0.046
smoking status	former current	294 (44.1%) 153 (22.9%)	97 (20.2%) 102 (21.2%)	< 0.001
Hypertension^a^		533 (73.9%)	356 (66.4%)	0.005
type 2 diabetes^a^		142 (19.5%)	73 (13.3%)	0.004
Statin therapy^b^		216 (29.7%)	91 (16.5%)	< 0.001
CHOL (mmol/l)		5.48 (1.10)	6.04 (1.04)	< 0.001
HDL-C (mmol/l)		1.38 (0.37)	1.74 (0.44)	< 0.001
LDL-C (mmol/l)		3.46 (0.99)	3.72 (0.94)	< 0.001
CPA_max (max. area, mm^2^)		27.2 (17.6)	19.6 (13.3)	< 0.001
CPA_sum (sum of plaque area, mm^2^)		56.4 (46.2)	34.8 (31.4)	< 0.001
CPA_mean (mean area of plaque, mm^2^)		18.0 (9.1)	14.5 (7.9)	< 0.001
CPS_max (max. stenosis, in %)		32 (19)	27 (17)	< 0.001
CPS_sum (sum of stenosis, in %)		71 (60)	50 (47)	< 0.001
CPS_mean (mean degree of stenosis, in %)		22 (11)	20 (11)	0.006
number of Carotid Plaques		2.9 (1.5)	2.2 (1.3)	< 0.001
Plaques at ACC	unilateral bilateral	160 (22.0%) 112 (15.4%)	95 (17.3%) 42 (7.6%)	< 0.001
Plaques at Bulbus	unilateral bilateral	232 (32.2%) 462 (64.1%)	250 (45.9%) 271 (49.7%)	< 0.001
Plaques at ACI	unilateral bilateral	225 (33.1%) 153 (22.5%)	132 (25.7%) 58 (11.3%)	< 0.001
cIMT mean		0.85 (0.16)	0.81 (0.14)	< 0.001
cIMT max		1.12 (0.27)	1.09 (0.24)	0.351

Binary / categorical variables are given in absolute counts (% per sex). Continuous variables are reported by mean (SD). Differences between sexes were tested with the Chi-squared test (categorical traits) or the Mann-Whitney U test (continuous traits). CPB statistics are given before transformation.

^a^ Known, in therapy or acute

^b^ ATC-code beginning with C10

We analyzed the correlation of CBP traits, cIMT and lipid parameters total, HDL, and LDL cholesterol. Considering all subjects, we detected a weak inverse correlation between CBPs and HDL cholesterol (spearman ρ about -0.10, see S2 Fig, S1 Table). Analyzing women only, the positive correlation with total and LDL cholesterol reached significance for three and four CBP traits, respectively. This correlation increased further when restricting to women without statin treatment (spearman ρ of all CPBs ranging from 0.11 to 0.21). There was a moderate positive correlation between mean cIMT and CPBs (spearman ρ between 0.21 and 0.36), which was stronger in men than in women. The correlation with maximal cIMT was weak, and not significant in the sex-separated analyses.

We tested the cardiovascular risk factors sex, age, BMI, smoking status, hypertension, type 2 diabetes, and statin therapy for effects on carotid plaque burden in all participants and separately in men and women (see S2 Table). BMI was weakly associated with the CPB traits, and this association was mainly driven by men. Of note, BMI had a negative effect on CPBs, e.g. a higher BMI is associated with smaller plaque area. Batagini et al. [46] also described lower BMI in patients with carotid artery stenosis compared to controls, which is in line with our findings for BMI and CPB. Type 2 diabetes was only univariately associated with traits considering the sum of plaques (area as well as stenosis). Although associated, all risk factors explained only small percentages of trait variance (e.g. 6% explained variance of CPS_mean, 19% of CPA_sum).

### Heritability of CPBs

Using a univariate model, we could not detect any significant heritability for the CPB traits (see S3 Table). After adjusting for all above mentioned cardiovascular risk factors, the heritability estimates increased and ranged between 68% (CPA_max, maximal area, p = 0.039) and 82% (CPS_max, maximal stenosis, p = 0.013). For our previously considered plaque score (PS) we detected significant estimates for both models (univariate model: h^2^ = 21%, p = 0.025; adjusted for risk factors: h^2^ = 33%, p = 0.002). Here, a larger sample size was available (N = 4,023).

### GWAS results

**Fig 1:**
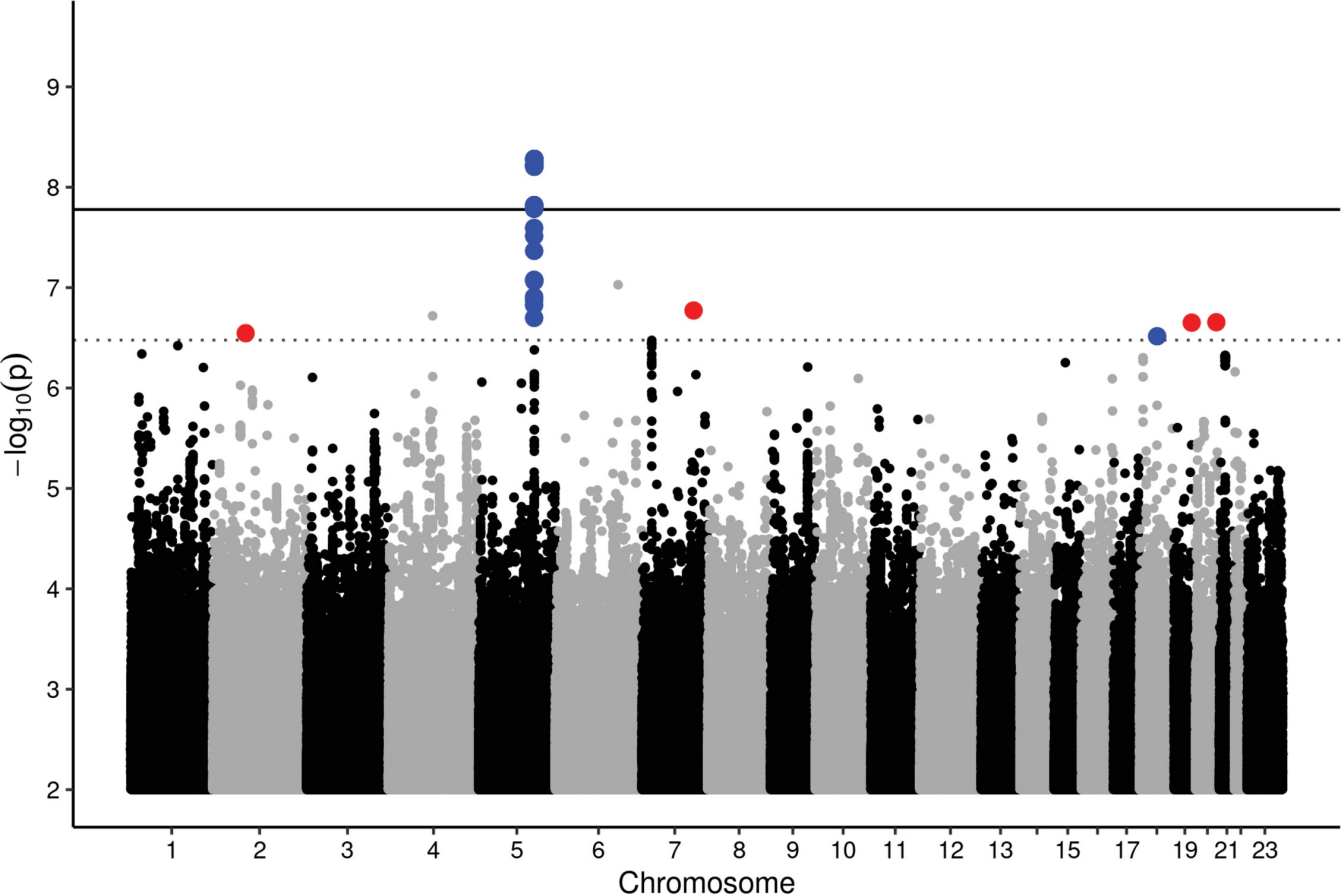
Manhattan-plot of all CPB traits. Minimal p-values across the six traits and three setting were displayed. The line corresponds to a genome-wide significance threshold of 1.67x10^-9^. Blue points mark regions associated strongest in men, red associations in women.

In our GWAS, we detected no signs for inflation of test statistics (λ ranged between 1.00 and 1.02). A Manhattan plot for all CPB traits is provided in Fig 1. After priority pruning with r^2^>0.1, we detected seven different loci reaching at least suggestive significance (*α_sug_* = 3.33×10^−7^) and one reaching genome-wide significance (*α_gw_* = 1.67×10^−8^). One of the suggestive loci was not supported in the regional association plot by any other variant and therefore excluded from further analysis. The remaining seven loci are presented in Table 2.

**Table 2:** Lead-SNPs of associated loci.

Cytoband	Lead SNP	Candidate	EA / OA	EAF	Info	associated CPBs	beta	SE	p-value	IA
		Genes								
5q31.1	rs201629990	*IL5* (78 kb)	GT / G	0.038	0.982	**CPA_mean** (A, **M**) CPA_max (A, M)	-0.401	0.068	5.22x10^-9^	6.85x10^-3^
6q21	rs72942431	*SELL* (trans-eQTL)	A / G	0.300	0.940	**CPS_max** (**A**) CPS_mean (A)	-0.151	0.029	5.36x10^-8^	0.466
4q22.3	rs3821968	*BMPR1B* (0 kb)	T / C	0.118	0.961	**CPS_sum** (**A**)	0.299	0.057	1.91x10^-7^	0.840
20q13.31	rs58056086	*TFAP2C* (42 kb)	T / C	0.088	0.887	**CPS_mean** (**W**) CPS_max (W)	-0.319	0.061	2.21x10^-7^	2.60x10^-4^
19q13.33	rs4539714	*FPR1* (989 kb)	C / T	0.194	0.905	**CPA_sum** (**W**)	0.360	0.069	2.23x10^-7^	4.70x10^-4^
2p12	rs6729048	*ABCC1* (trans eQTL)	A / T	0.137	0.995	**CPA_sum** (**W**)	-0.369	0.071	2.84x10^-7^	2.19x10^-3^
18q12.3	rs7245281	*PSTPIP2* (160 kb)	A / G	0.197	0.956	**CPS_sum** (**M**)	-0.322	0.062	3.05x10^-7^	2.00x10^-5^

Per locus the most strongly associated variant is given. Beta estimate, standard error (SE) and p-value are reported for the best-associated phenotype and modus (marked bold). Biologically meaningful genes are listed as candidates (plausible in context with atherosclersosis and sex-specifity). For cis-eQTLs distance in kb is given. For statistics of the other CPBs and full annotation, see S4–S7 Tables. Abbreviations: EA/OA: effect/other allele; EAF: effect allele frequency; info: imputation info score; IA: p-value of difference test.

The strongest association was detected for CPA_mean with the SNP rs201629990 at cytoband 5q31.1 in men (β = -0.401, *p* = 5.22x10^-9^). In the combined analysis, the association reached only suggestive significance (p = 1.19x10^-7^), but the SNP was not associated in women (p = 0.093). The variant was also associated with CPA_max at suggestive significance in men and the combined setting. It is a non-coding exon modifier of a Y-RNA, which are known to induce inflammation and an atherogenic effect was described [47,48]. It is also an intron modifier of *C5orf56*, which codes for an Interferon regulatory factor 1 (IRF1) antisense RNA. The lead SNP is supported by 29 variants in LD (r^2^>0.1) and is an eQTN or in LD with an eQTLn for 23 genes including the aforementioned genes *IRF1* [29] and *C5orf56* [28] (LD r^2^>0.3). Other GWAS previously reported “Red cell distribution width” [49], fibrinogen levels [50–52], and chronic inflammatory diseases [53] for this region (LD r^2^>0.75). Summary statistics, nearby genes, eQTL and GWAS Catalog look-up are provided in S4–S7 Tables, respectively.

### Comparison of models

Using different models (univariate association, adjusting for up to 10 genetic PCs, adjusting for cardiovascular risk factors) did not significantly change the effect sizes (S8 Table). In a multivariate model including all seven top-SNPs, the explained variance by genetics was about 6% in men and 12% in women. Adding cardiovascular risk factors, the explained variance increased to similar values in men and women (e.g. CPA_mean with 10.7% and 12.5% in men and women, respectively). In all combined models, almost all SNPs remained significant, although no longer on suggestive but nominal level (see S8 Table).

**Fig 2:**
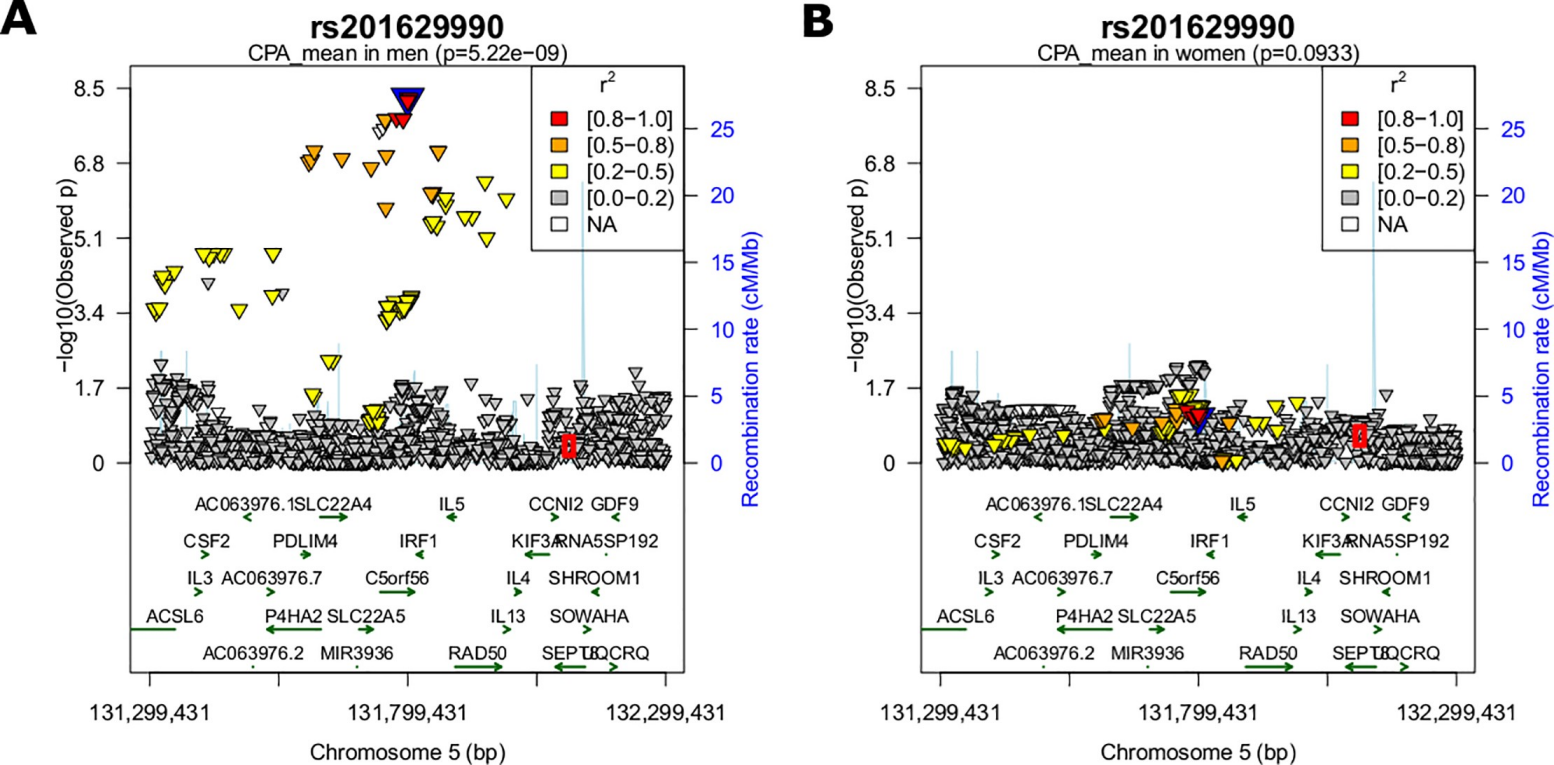
Regional association plots. Regional association plot of locus 5q31.1 for (A) men and (B) women. P-value of SNPs were log10 transformed and colored by LD (r^2^ to lead SNP rs201629990). The red circle marks the position of the independent hit detected in the credible set analysis in women, rs33967116. This SNP is nominally associated with carotid plaque score (see S4 Fig).

**Fig 3:**
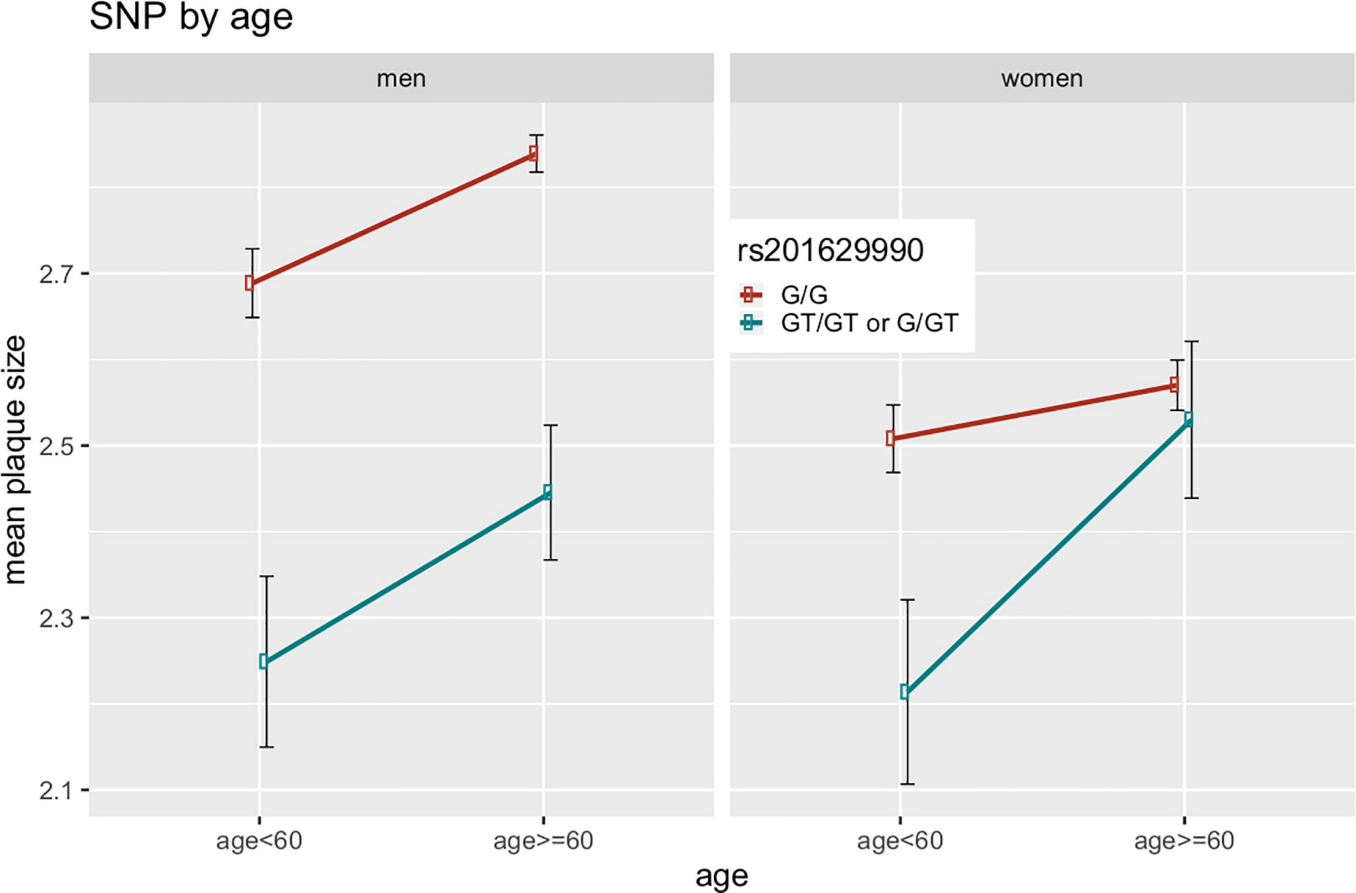
SNP-by-sex interaction plots. Interaction plot of Top-SNP rs201629990 by age (younger than 60 years, older than 60 years) for men and women. Since there were only two participants with genotype “GT/GT”, we pooled them with the genotype group “GT/G” (n = 108 for both “GT/GT” & “GT/G”, n = 1169 for”G/G”).

Testing for sex interaction, we compared the effect sizes calculated in the sex-stratified analyses. Here, we found significant difference in effect size for all sex-specific loci. We repeated this analysis for all associated SNPs at the top locus 5q31.1 and found SNPs in high LD with the lead SNP (r^2^>0.6) showing the same behavior. Less correlated variants showed no sex-specificity (see regional association plots for men and women in Fig 2 and S9 Table). Since estrogen-response elements were reported for this region, we tested whether this is an age-dependent phenomenon, e.g. menopausal status in women. For participants younger than 60 years, we detected no significant difference in effect sizes, but within the elderly, 29 SNPs showed significantly stronger effects in men than women (see Fig 3 for SNP-age interaction).

We performed joint analyses of 2,279 SNPs at this locus (+/- 500 kb of lead SNP rs201629990) for all samples, and separately for men and women. Each run produced one independent hit: rs114597978 for all (LD to rs201629990, r^2^ = 0.929), rs201629990 in men and rs33967116 in women (LD to lead SNP r^2^ = 0.003). All 24 associated SNPs generate an 82.7% credible set in the combined analysis, whereas in the subset of men, those SNPs produced a 91.9% credible set (S3 Fig). They had all shown a male-specific effect. In contrast, the 91.9% credible set for women contained 1903 SNPs due to lack of a strong signal. In men, the lead SNP was ranked third with a posterior probability of 0.063. In summary, the variant causing the association is most certainly one of the male-specific SNPs. However, we cannot use the credible set to reduce the SNP list of our GWAS, as all associated SNP are within the 95% credible set.

We repeated our analyses of this locus with the carotid plaque score PS, which we described in a previous work [7]. The region was nominally associated with PS, with lead SNP rs339671116 (p<10^−5^, see S4 Fig and S10 Table). Of note, this is the same SNP as detected as a single independent hit in women. However, this SNP does not display any sex-interaction. We repeated our credible set analyses conditioned on rs339671116, but the sets did not change, suggesting that rs201629990 and rs339671116 correspond to separate signals.

### Gene expression lookup for 5q31.1

Since 5q31.1 displayed male-specific effects on CPA_mean, we created a candidate list of 29 nearby genes and 24 eQTL genes of all 24 SNPs in the 95% credible set (total n = 40 candidate genes). We could match 32 of them to high quality gene-expression probes including probes mapping to the same gene.

We tested these 32 probes for association with CPA_mean in all samples and in men and women separately. For men, we detected an association with IL5 and CCNI2. In women, GDF9 and P4HA2 reached nominal significance (see Table 3). However, these results were not robust to multiple testing. Comparing the effect sizes of those five genes in men and women, IL5 had a significantly greater effect size in men than in women (interaction *p* = 0.037). According to the GTex database, there are no eQTLs for IL5 probably due to a low expression except for testicular tissue (see S11 Table and S5 Fig).

**Table 3:** Probes associated with CPA_mean.

INFO		COMBINED		MEN		WOMEN		Interaction	
Probe	Gene	Beta	P	Beta	P	Beta	P	Diff.	P
ILMN_1709300	IL5	0.537	0.102	1.117	0.007	-0.297	0.581	1.414	0.037
ILMN_2184575	CCNI2	-0.555	0.080	-1.028	0.011	0.047	0.927	-1.074	0.097
ILMN_1738783	GDF9	0.237	0.502	-0.517	0.265	1.161	0.031	-1.677	0.018
ILMN_2381697	P4HA2	0.556	0.154	0.069	0.890	1.289	0.040	-1.220	0.126
ILMN_1730025	ACSL6	-0.627	0.082	-0.924	0.045	-0.136	0.814	-0.788	0.286

Association was calculated under adjustment for percentages of lymphocytes, monocytes, and age. The combined setting was additionally adjusted for sex. The first two probes reached Bonferroni-adjusted significance (α = 0.0167 adjusting for three tests). For interaction analyses, effect sizes for men and women were compared.

### CAD and cIMT look-up

We successfully verified six carotid plaque or cIMT SNPs and one CAD SNP (see S12 and S13 Tables) for association with Plaque burden. For the carotid SNPs, we did not detect more SNPs than would be expected by chance (binomial test, p = 0.045) and for CAD, we found less SNPs than expected by chance (binomial test, p = 0.005).

The PGS was only associated with CPS_max in the combined setting (see S14 Table). Interestingly, PGS was not associated with any CPB trait in men, but with all traits in women (α = 0.05). We performed a difference test on the sex-specific PGS effects, and found for both CPA_max and CPS_max significant female-specific effects. We checked our results with the aforementioned score PS (n = 6639; 3198 men & 3441 women), and found a similar trend: in women the association was more significant and the effect size was greater. However, the difference test here was not significant.

## Discussion

Carotid plaque burden is an understudied subclinical trait of atherosclerosis due to its time-consuming and complicated assessment. Indeed, people at high risk for cardiovascular events can be identified by carotid ultrasound and steps for prevention be taken. Carotid plaque burden can then be used to control the progress of plaque formation.

To the best of our knowledge, there is only one other small GWAS in people of Hispanic ancestry that analyzed carotid plaque area similar to our trait CPA_sum [15] with focus on smoking interaction. However, no genome-wide significant associations were reported there. Atherosclerosis is known for its sexual dimorphism, i.e. men have a higher risk for coronary artery disease than women [9]. However, genetic reasons have not been described so far [8]. Here, we conducted a genome-wide study of CPB traits and compared the effect sizes of suggestive genetic loci for sex-interactions.

We detected an association at 5q31.1 with CPA_mean and CPA_max at genome-wide significance in men. Our sex-interaction analyses showed a significant difference in effect sizes for men and women. Chen et al. [54] had also analyzed this genetic region in context of schizophrenia, where they also detected male-specific associations. As a possible explanation, they named estrogen-response elements in this region, which would be in line with recent findings of steroid hormones influencing the risk of coronary artery disease [11].

The locus is rich in genes and we identified 23 candidates from our secondary analyses comprising credible set analysis, nearby gene annotation, eQTL look up and gene-expression association analysis. The most plausible candidate is *IL5*, whose gene-expression displayed the same male-specific effect on CPA_mean. IL5 is released by mast cells, which are involved in the development of many diseases [55]. Silveira et al. [56] had analyzed plasma IL5 and its effect on cIMT, and had found a protective effect of IL5 for women. However, since the gene expression association is not robust for multiple testing, we cannot completely rule out that other genes are responsible for the observed genetic association: the lead SNP is an eQTL of *IRF1* antisense RNA. *IRF1* expression is downregulated by estradiol [57] and reported for being associated with fibrinogen levels [50–52], which is a known risk factor for atherosclerosis in carotid arteries [46,58]. Also the *Y-RNA*, in which the lead SNP is located, is a potential candidate, as it is known to induce inflammation and to have a atherogenic effect [47,48].

We detected six other hits with suggestive significance, which were in or nearby plausible genes. The hit at 6q21 was in LD (r^2^ = 0.35) with a trans-eQTL of *SELL* [31], coding for l-selectin. Low levels of l-selectin were shown to be associated with carotid artery plaque size [59]. We detected an independent locus at 4q22.3 near *BMPR1B*, which is a receptor necessary for the signal transduction of BMP2–Smad1/5/8 signaling pathway, known for its association with vascular calcification [60]. Common genetic variants within *BMPR1B* have been reported for association with cIMT [61]. Further sex-specific hits were at 20q13.31 (associated in women, candidate gene *TFAP2C*, coding for estrogen receptor 1), 19q13.33 (associated in women, candidate gene *FPR1*, coding for a receptor for N-formyl-methionyl peptides. These neutrophil chemotactic factors activate polymorphonuclear neutrophils, which contribute to the pathogenesis and progression of atherosclerosis [62]), 2p12 (associated in women, candidate gene *ABCC1*, coding for an ATP-binding cassette, which are involved in cholesterol homeostasis and vascular inflammation [63], and 18q12.3 (associated in men, candidate gene *PSTPIP2*, which has been detected as differentially expressed gene in the atherosclerosis [64]). However, these associations require validation in larger studies or meta-analyses.

Previous family-studies estimated heritability of carotid plaque area at 69.1% (adjusted for sex and age) [65] and of carotid plaque prevalence at 50% (adj. for age, hypertension, diabetes, smoking, BMI, and WHR) [66]. We estimated the heritability using unrelated samples, and achieved similar results: adjusted for sex and age, we estimated about 49.7% and adjusted for all cardiovascular risk factors 70.5%. This increase can be explained by the reduction of noise: after adjustment on risk factors, 70.5% of the remaining variance can be explained by genetics.

Using our seven independent SNPs, we could explain about 6–12% of the CPBs variance, depending mainly on sex: for all traits the explained variance in women was larger than in men. This is complementary to the non-genetic model, in which the cardiovascular risk factors explained more variance in men than in women. Using the polygenic score for CAD [9], we found similar results, as the score was only associated in women, explaining about 1% variance. These results suggest that the genetic influence on atherosclerosis is stronger in women, whereas non-genetic factors like BMI are more relevant for men.

Interestingly, the same SNPs in the same multivariate model explained more variance for men than for women, although only one SNP showed a significant sex-interaction. This still applies when rs114597978 was removed from the model as a sensitivity test.

Our previously published study on genetics of carotid plaque showed significant enrichment of CAD loci with carotid plaques [7], and other studies revealed a genetic correlation of carotid plaques, cIMT, coronary heart disease and stroke [6]. However, we could not detect such an enrichment for our CPB traits. Regarding CAD SNPs, we even detected less significant variants than expected by chance. We also tested for an enrichment of cIMT and carotid plaque loci in our data, but again we found not more than expected by chance. This suggests a different genetic mechanism for plaque formation and its growth. This is in line with findings of Zeller et al. [13], who could only confirm one CAD locus for CAD severity. The same was observed for the risk of myocardial infarction in stable CAD patients, where significant differences in the effects on CAD onset and MI were found (personal communication).

A major limitation of our study is the small sample size for genome-wide analyses caused by the fact that the phenotype is difficult to obtain. This issue is even more severe for the sex-stratified analyses. Therefore, replication of our GWAS results in independent cohorts is necessary.

In conclusion, we analyzed traits of carotid plaque burden and detected a genome-wide significant hit at 5q31.1 for men only. The locus includes several estrogen response elements, suggesting a new link between steroid hormone regulation and atherosclerosis. However, verification of the causal gene at this region requires further analysis, taking gene-expression and estrogen regulation into account. Using all suggestive hits, we could explain 10% of the CPBs variance, while our estimated heritability was about 35%, suggesting genetic loci still undetected. Larger studies and meta-analyses are required to verify the suggested loci and to unravel the underlying molecular mechanisms.

## Supporting information

S1 Table Correlation results. For all analyses, spearman rank was calculated. P-values are given in the upper triangle, Spearmans rho in the lower triangle. Significant p-values are marked in red (p<0.05).
(XLSX)

S2 Table Univariate & multivariate models of cardiovascular risk factors on CPBs. Significant p-values are marked in red (p<0.05).
(XLSX)

S3 Table GCTA heritability estimation. For all six CPB traits, we estimated the heritability (h2) without any adjustment (univariate), with adjustment for sex and age (as in GWAS), and with adjustment for sex, age, BMI, hypertension, statin treatment, amoking and type 2 diabetes. Significant p-values are marked in red (p<0.05). Yellow marked p-values indicated a trend (p<0.1).
(XLSX)

S4 Table Summary statistics of the nine suggestive significant loci of GWAS (adjusted for sex and age). Lead SNPs are marked green in column A. Suggestive significant associations are marked red in column M (p<3.33x10e-7). Results for all CPBs are given at the end.
(XLSX)

S5 Table Nearby genes of the associated SNPs. Lead SNPs are marked green in column A.
(XLSX)

S6 Table eQTL lookup of the associated SNPs. Lead SNPs are marked green in column A.
(XLSX)

S7 Table GWAS Catalog lookup of the nine suggestive loci. Lead SNPs are marked green in column A.
(XLSX)

S8 Table **Comparison of different models for all subjects (a, d, g), male subjects (b, e, h) and female subjects (c, f, i).** In Tables a-c, single SNPs results are shown (CPB ~ SNP + …), with respect only to the top phenotype per SNP. In Tables d-f, all SNPs were analyzed together (CPB ~ SNP1 + SNP2 +… + SNP9), and in Tables g-i with additional adjustment for cardiovascular risk factors (CPB ~ SNP1 +…+ SNP9 + CV risk factors). Here, all traits were analyzed. Suggestive significant p-values are marked red (p<1E-6) and nominal sigificant p-values are marked yellow (p<0.05).
(XLSX)

S9 Table Sex-Interaction results. Table a) We compared SNP effect sizes of men and women for all lead SNPs. Only the difference of effect of rs114597978 (cytoband 5q31.1) reached significance. Table b) We compared effect sizes of all SNPs at 5q31.1 for sex-interaction, sex-interaction within the elderly (older than 60 years) and interaction regarding statin treatment (in men only). Significant p-values are marked red (p<0.05).
(XLSX)

S10 Table Association look-up for PS. We listed all SNPs associated with PS or CPA_MEAN at at least suggestive level (p<1E-6). Association statistics of PS are taken from our previous publication (Pott et al. Genome-wide meta-analysis identifies novel loci of plaque burden in carotid artery. Atherosclerosis, 2017) and their newly analzed subgroups for men and women. There is no significant interaction for PS.
(XLSX)

S11 Table Gene expression results. Associations of all analyzed probes are shown.
(XLSX)

S12 Table Candidate look-up of 159 CAD SNPs. (XLSX)

S13 Table Candidate look-up of 141 CIMT and carotid plaque SNPs. (XLSX)

S14 Table Association results of polygenetic score (PGS) of coronary artery disease (CAD) and the carotid plaque traits. Significant results are marked in red (α = 0.05). Sample sizes are 1277 (757 men + 550 women) for the burden traits (CPAs and CPS) and 6639 (3198 men + 3441 women) for the plaque score PS.
(XLSX)

S1 Fig QQ-Plots for all CPB traits. (PDF)

S2 Fig Correlation Plots for CBP traits and CV risk factors (all vs men vs women). (PDF)

S3 Fig Credible Sets at 5q31.1. (PDF)

S4 Fig RA Plots of 5q31.1 for CPA_MEAN and PS. (PDF)

S5 Fig Interaction Plot of IL5 blood gene-expression. (PDF)
