# Supplementary material for: Genome-wide analysis of carotid plaque burden suggests a role of IL5 in men
Source: PLoS One. 2020 May 29;15(5):e0233728. doi: 10.1371/journal.pone.0233728 (PMC7259763; doi:10.1371/journal.pone.0233728)
Supplement: S1 Fig — (PDF) [file pone.0233728.s015.pdf]

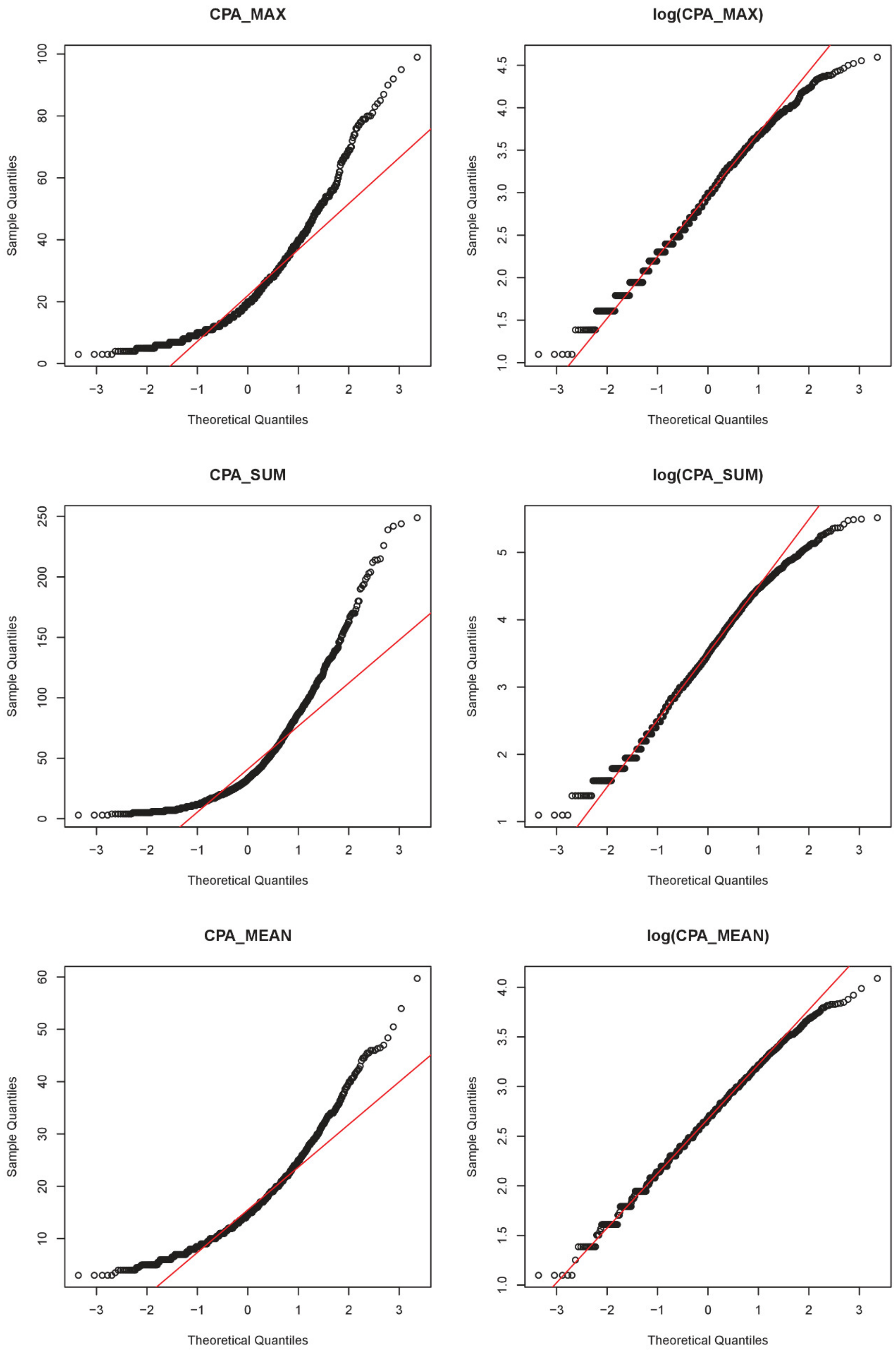

Figure S1-A: QQ-Plots for CPA traits. The left side shows the distribution before transformation, on the right side the distribution after log transformation is given. After transformation, data is close to normal distributed.

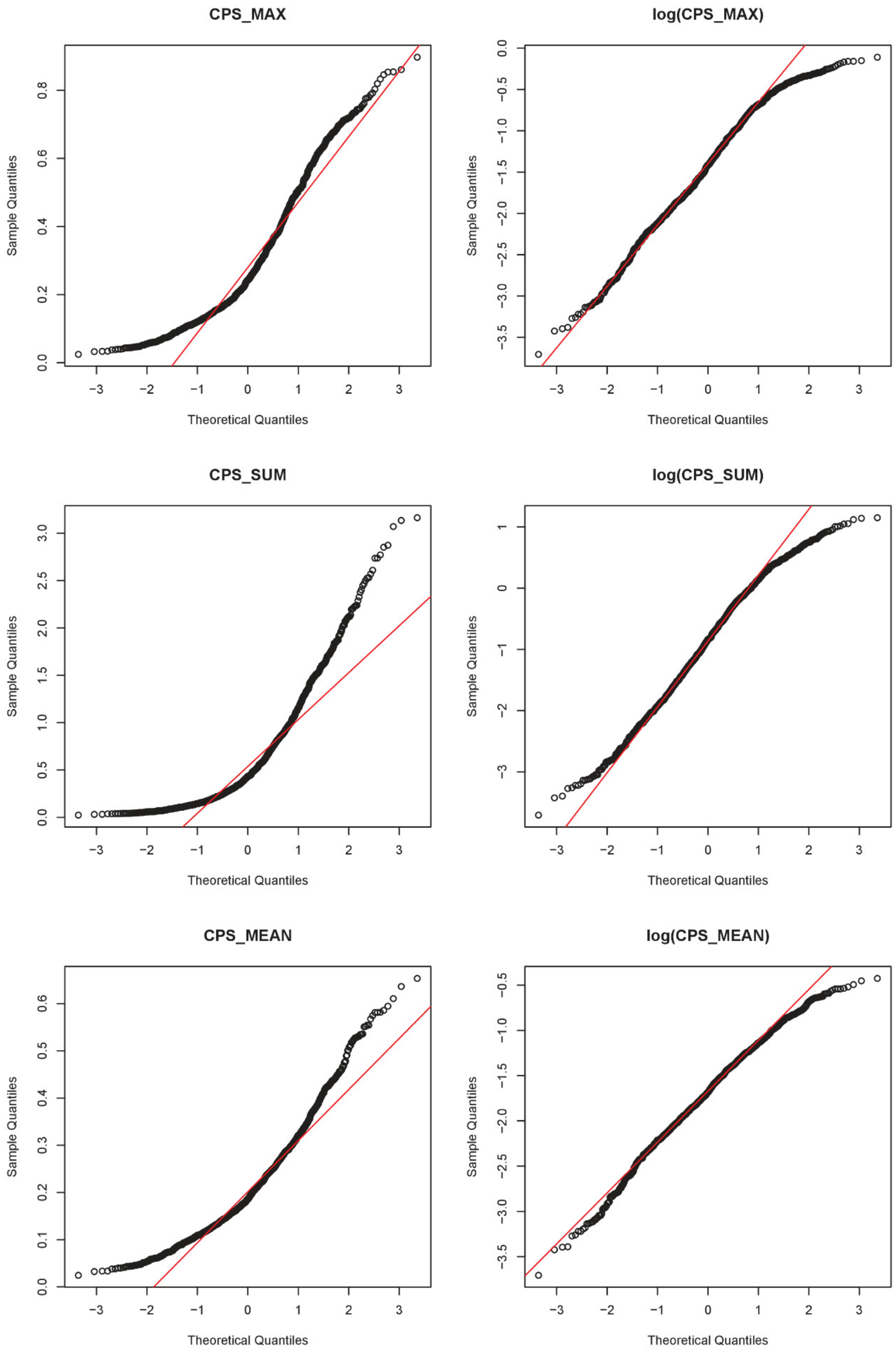

Figure S1-B: QQ-Plots for CPS traits. The left side shows the distribution before transformation, on the right side the distribution after log transformation is given. After transformation, data is close to normal distributed.
