# Supplementary figures and images for: Genome-wide analysis of carotid plaque burden suggests a role of IL5 in men
Source: PLoS One. 2020 May 29;15(5):e0233728. doi: 10.1371/journal.pone.0233728 (PMC7259763; doi:10.1371/journal.pone.0233728)

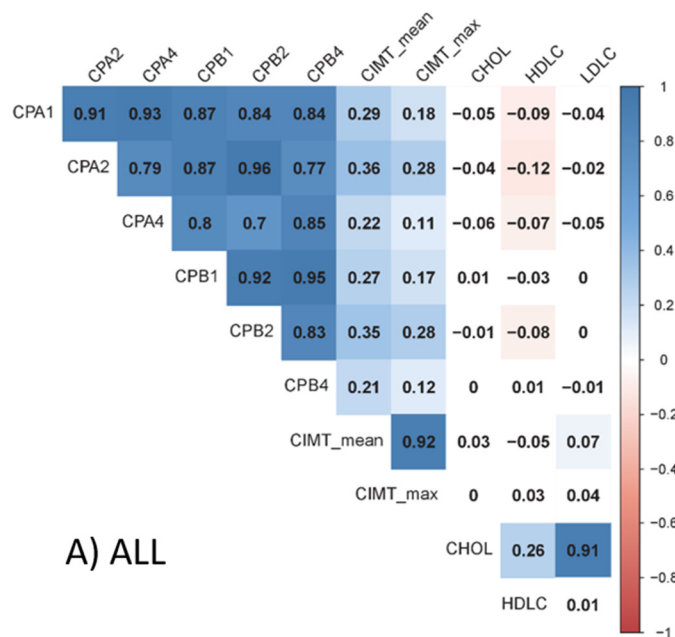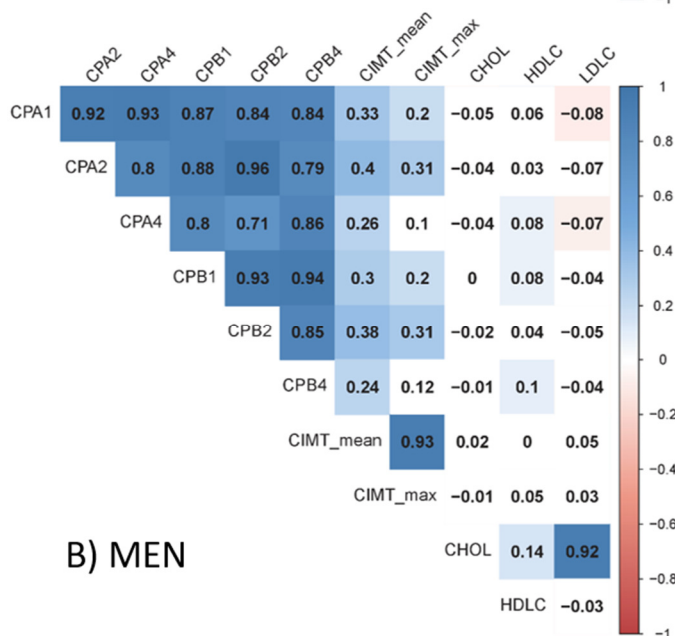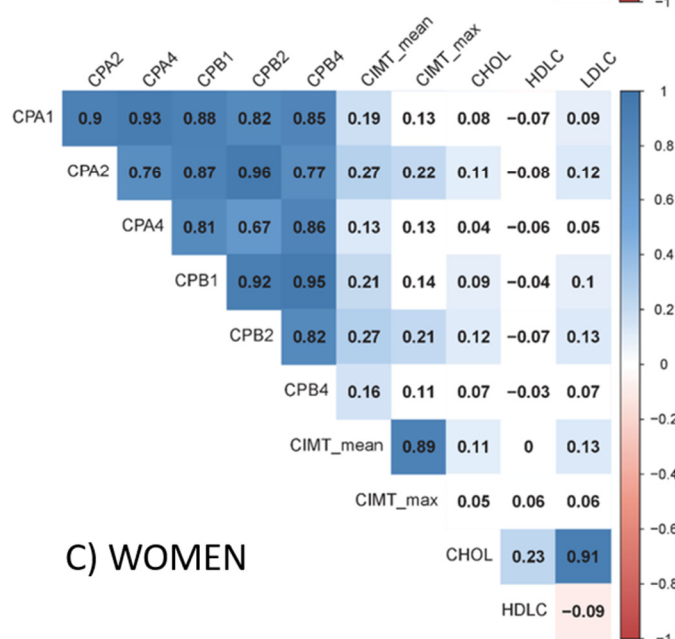

Supplement: S2 Fig — (PDF) [file pone.0233728.s016.pdf]
