# Supplementary material for: Genome-wide analysis of carotid plaque burden suggests a role of IL5 in men
Source: PLoS One. 2020 May 29;15(5):e0233728. doi: 10.1371/journal.pone.0233728 (PMC7259763; doi:10.1371/journal.pone.0233728)
Supplement: S3 Fig — (PDF) [file pone.0233728.s017.pdf]

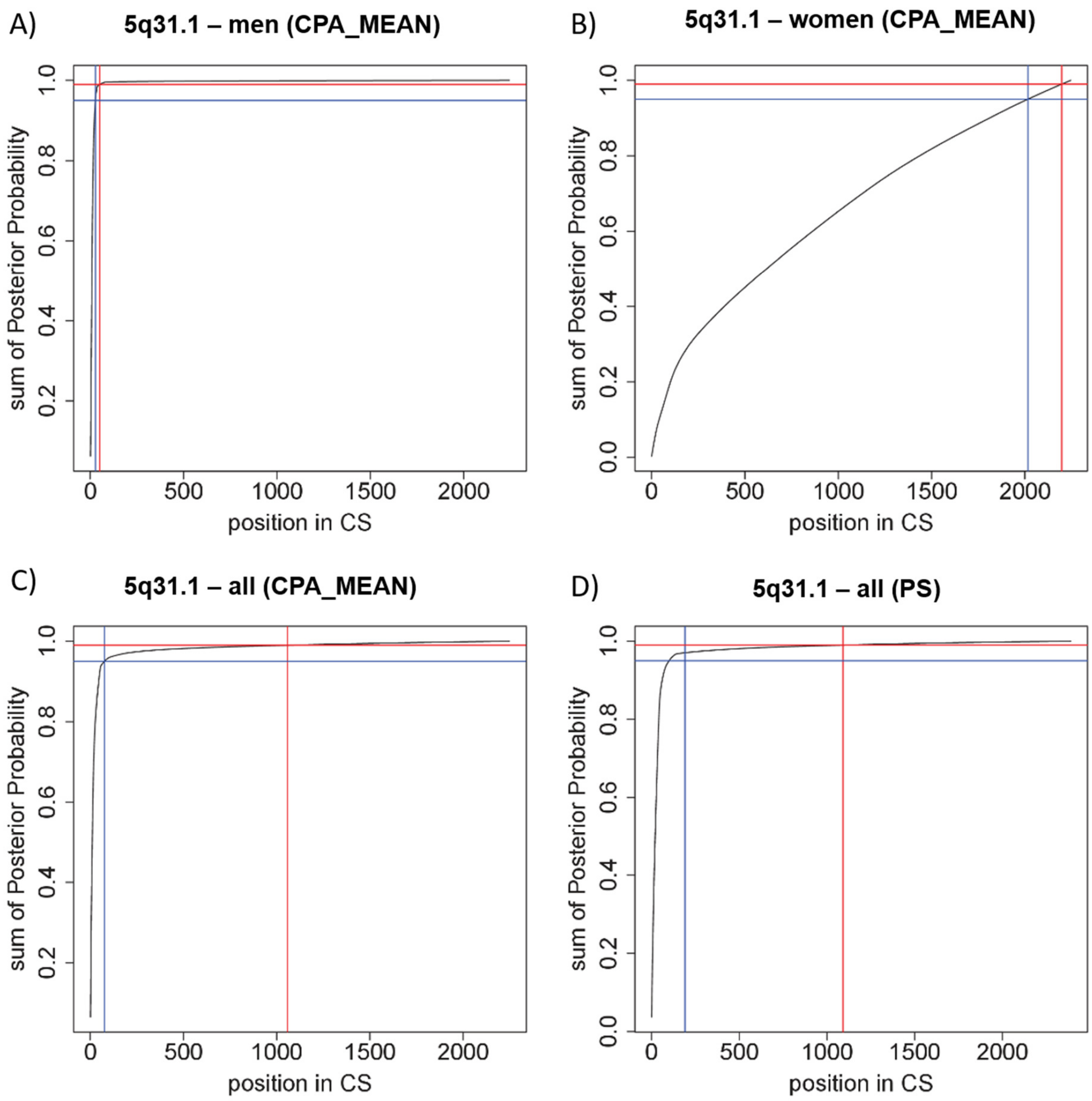


*Figure S3: Results of credible set (CS) analyses of 2250 SNPs around lead SNP rs114597978 +/- 1000 kb. SNPs were ordered by their posterior probability. Blue lines mark the number of SNPs within the 95% CS, red lines the 99% CS. A) For men, 28 SNP were within the 95% CS, and 51 within the 99% CS. B) For women, the 95% CS consisted of 2016 SNPs (99% CS: 2197 SNPs). C) Using all subjects, 76 SNPs were within the 95% CS (99% CS: 1058 SNPs. D) Using the carotid plaque score PS instead of CPA_MEAN as dependent variable, the 95% CS consisted of 101 SNPs (99% CS: 1092).*
