# Supplementary material for: Genome-wide analysis of carotid plaque burden suggests a role of IL5 in men
Source: PLoS One. 2020 May 29;15(5):e0233728. doi: 10.1371/journal.pone.0233728 (PMC7259763; doi:10.1371/journal.pone.0233728)
Supplement: S4 Fig — (PDF) [file pone.0233728.s018.pdf]

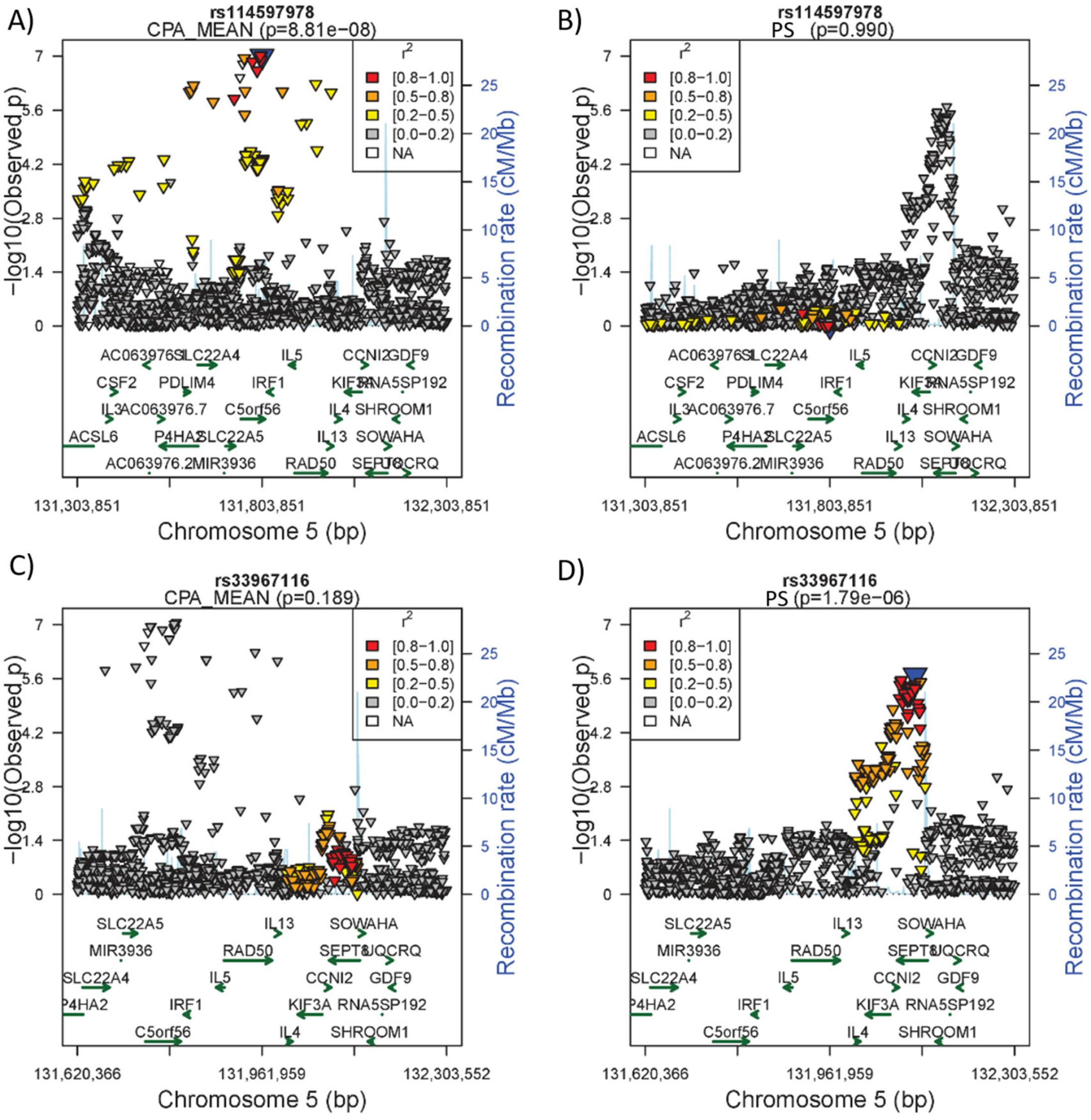

Figure S4: Regional association (RA) plots of 5q31.1. SNPs are colored according to their LD with the lead SNP (Panel A & B: rs114597978; Panel C & D: rs33967116; using 1000 Genomes Phase 3, Europeans only). P-Values are shown for the respective trait (Panel A & C: CPA_MEAN; Panel B & D: carotid plaque score PS). As one can see, the two signals are specific for the two traits.
