# Supplementary material for: Genome-wide analysis of carotid plaque burden suggests a role of IL5 in men
Source: PLoS One. 2020 May 29;15(5):e0233728. doi: 10.1371/journal.pone.0233728 (PMC7259763; doi:10.1371/journal.pone.0233728)
Supplement: S5 Fig — (PDF) [file pone.0233728.s019.pdf]

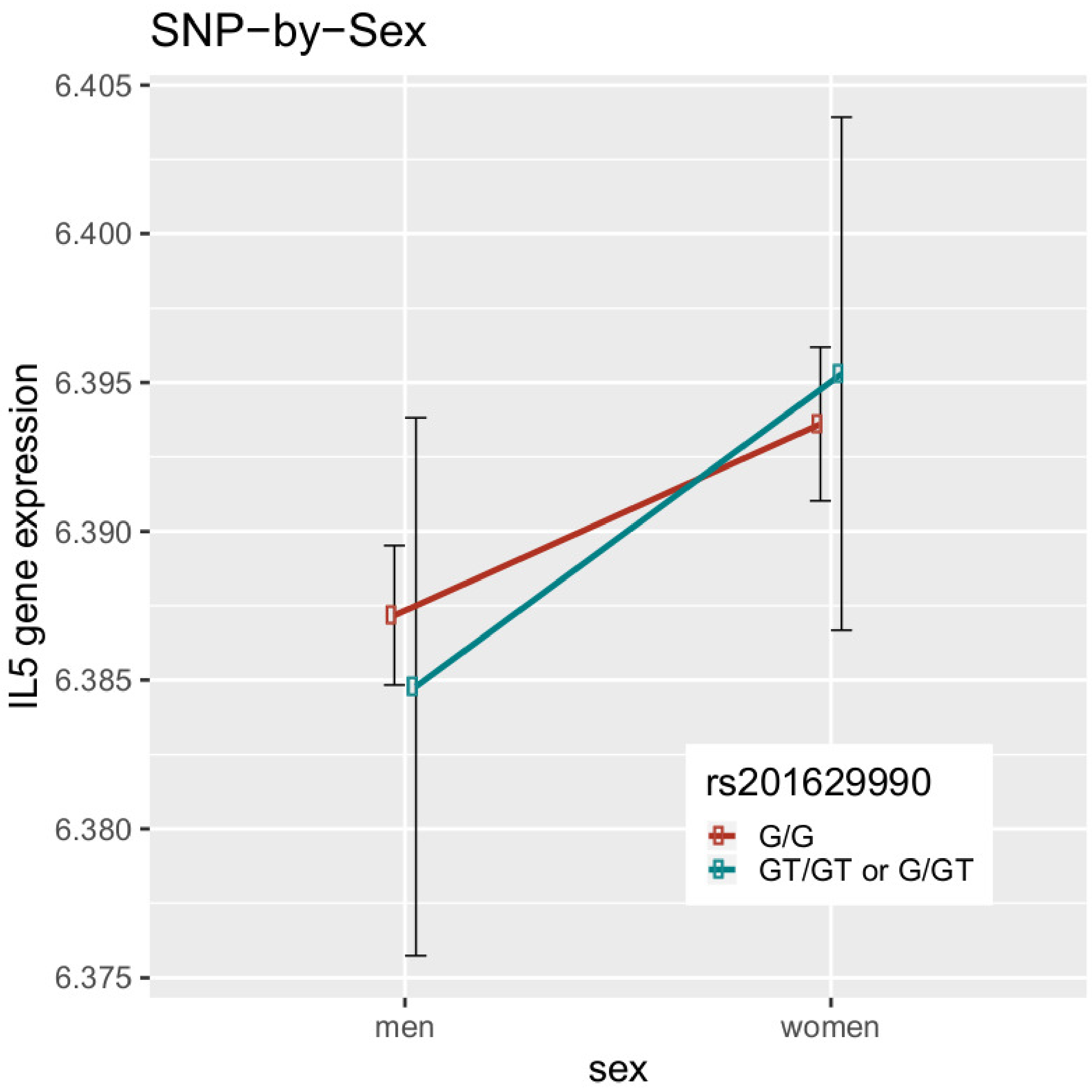

Figure S5: Interaction plot for IL5 blood gene-expression levels by sex and genotype (lead SNP rs201629990). In women, the insertion increases IL5 expression levels, while in men it decreases the gene-expression.
